# Complete Genome Sequence of Peste des Petits Ruminants Virus from Georgia, 2016

**DOI:** 10.1128/genomeA.01091-17

**Published:** 2017-10-12

**Authors:** Paulina Z. Rajko-Nenow, Tabitha G. Cunliffe, John T. Flannery, Honorata M. Ropiak, Lasha Avaliani, Marina Donduashvili, Michael D. Baron, Carrie A. Batten

**Affiliations:** a The Pirbright Institute, Pirbright, Woking, Surrey, United Kingdom; b LEPL National Food Agency of Georgia, Tbilisi, Georgia; c Laboratory of Ministry of Agriculture of Georgia, Tbilisi, Georgia

## Abstract

We report here the complete genome sequence of a peste des petits ruminants virus (PPRV) from the first outbreak of the disease in Georgia in January 2016. Genome sequencing was performed using Illumina next-generation sequencing technology in conjunction with Sanger sequencing. This PPRV/Georgia/Tbilisi/2016 genome sequence clustered within lineage IV PPRV viruses.

## GENOME ANNOUNCEMENT

Peste des petits ruminants (PPR) is a highly contagious disease of sheep and goats caused by peste des petits ruminants virus (PPRV) (1). It has spread at an alarming rate in the past two decades, reaching many countries previously not affected. PPR causes significant annual losses (~$1.4 billion) attributed to decreased production and animal deaths, as well as the cost required to overcome the disease. Significant economic losses are incurred mainly by low-income livestock keepers in Africa, the Middle East, and Asia. As a result, the disease has become the target of a global eradication campaign led by the Food and Agriculture Organization of the United Nations (FAO) and the World Organisation for Animal Health (OIE) (2).

PPRV belongs to the genus *Morbillivirus* of the family *Paramyxoviridae* and is an enveloped, negative-sense, single-stranded RNA virus with a single serotype. The PPRV genome is 15,948 nucleotides (nt) in length (3), but longer variants were recently sequenced in outbreaks from China (4). Four distinct lineages of PPRV (I to IV) have been identified which correspond to the geographical distribution of the virus (5).

In January 2016, clinical signs, such as diarrhea and lesions in the mouth, tongue, gingiva, and nostrils, as well as a high mortality rate, were observed in sheep 1 to 2 months of age near Tbilisi, Georgia. The disease was initially suspected to be caused by bluetongue virus (BTV) (6); however, when specimens were submitted for confirmation to the EU Reference Laboratory for BTV at the Pirbright Institute, UK, the samples tested negative for BTV. PPRV was detected by real-time reverse transcription-PCR (RT-PCR) which confirmed PPRV to be the causative agent of disease in this instance.

The PPRV was isolated from lung tissue after two passages in Vero dog signaling lymphocyte activation molecule (SLAM) cells and named Georgia/Tbilisi/2016. Total RNA was extracted from the infected cell culture supernatant using the Direct-zol RNA miniprep kit (Zymo Research). Library preparation was performed using the Nextera XT DNA preparation kit (Illumina) and sequenced on the MiSeq platform. Reads were filtered with sickle and aligned to the reference genome using the bowtie2 and bwa tools. Upon PPRV genome assembly, an 88-bp gap was identified in the untranslated region between the coding sequences for the M and F proteins, a region of high GC content. This region was amplified using Kapa HiFi polymerase (Kapa Biosystems), and the PCR products were sequenced with an ABI 3730xl genome sequencer (Applied Biosystems). The generated sequences were assembled using the SeqMan Pro 13 software (DNAStar, Inc.).

PPRV Georgia/Tbilisi/2016 was 15,948 nucleotides in length, with a genome organization identical to that of its nearest relative, Ethiopia/2010 (GenBank accession no. KJ867541), with the two strains differing at 63 amino acid positions in the coding regions.

At the nucleotide level, Georgia/Tbilisi/2016 shared the highest identity with lineage IV strains, 98% with Ethiopia/2010 (accession no. KJ867541), 97% with Algeria/2015 (accession no. KY885100), and 97% with Morocco/2008 (accession no. KC594074); 96% with Turkey/2000 (accession no. AJ849636) and India/2015 (accession no. KT860064); and last, 94% with a recent strain from China/2015 (accession no. KT633939). Based on the full-genome sequences currently (as of August 2017) available in GenBank, the Georgia/Tbilisi/2016 strain is more closely related to recent North African isolates than recent PPRV strains from central Asia.

### Accession number(s).

The full-genome sequence of Georgia/Tbilisi/2016 has been deposited in GenBank under accession number MF737202.
